# Temozolomide and Radiotherapy versus Radiotherapy Alone in High Grade Gliomas: A Very Long Term Comparative Study and Literature Review

**DOI:** 10.1155/2015/620643

**Published:** 2015-03-01

**Authors:** Salvatore Parisi, Pietro Corsa, Arcangela Raguso, Antonio Perrone, Sabrina Cossa, Tindara Munafò, Gerardo Sanpaolo, Elisa Donno, Maria Antonietta Clemente, Michele Piombino, Federico Parisi, Guido Valle

**Affiliations:** ^1^Unit of Radiation Therapy of IRCCS “Casa Sollievo della Sofferenza”, 71013 San Giovanni Rotondo, Italy; ^2^Radiotherapy Unit, Scientific Institute “Casa Sollievo della Sofferenza”, Viale Cappuccini, 71013 San Giovanni Rotondo, Italy; ^3^Unit of Radiation Therapy of Policlinico, 70124 Bari, Italy; ^4^University of Chieti, 66100 Chieti, Italy; ^5^Unit of Nuclear Medicine of IRCCS “Casa Sollievo della Sofferenza”, San Giovanni Rotondo, Italy

## Abstract

Temozolomide (TMZ) is the first line drug in the care of high grade gliomas. The combined treatment of TMZ plus radiotherapy is more effective in the care of brain gliomas then radiotherapy alone. Aim of this report is a survival comparison, on a long time (>10 years) span, of glioma patients treated with radiotherapy alone and with radiotherapy + TMZ. *Materials and Methods*. In this report we retrospectively reviewed the outcome of 128 consecutive pts with diagnosis of high grade gliomas referred to our institutions from April 1994 to November 2001. The first 64 pts were treated with RT alone and the other 64 with a combination of RT and adjuvant or concomitant TMZ. *Results*. Grade 3 (G3) haematological toxicity was recorded in 6 (9%) of 64 pts treated with RT and TMZ. No G4 haematological toxicity was observed. Age, histology, and administration of TMZ were statistically significant prognostic factors associated with 2 years overall survival (OS). PFS was for GBM 9 months, for AA 11. *Conclusions*. The combination of RT and TMZ improves long term survival in glioma patients. Our results confirm the superiority of the combination on a long time basis.

## 1. Introduction

Despite advances in the last years in the treatment of neoplastic diseases, the prognosis of patients (pts) with high grade gliomas is still dismal.

The survival of glioma patients treated with surgical resection alone is approximately 6 months [1]. The combination of surgery and postoperative radiation therapy (RT) increases the survival up to 9-10 months in pts with glioblastoma multiforme (GBM) and 36 months in anaplastic astrocytoma (AA) [2].

In order to get further improvements in the last decades many studies have tested multimodality treatment schedules incorporating chemotherapy (CT) with nitrosourea based regimens, with questionable survival advantages [1–6].

A meta-analysis published in 2002 [7] including many different chemotherapeutic regimes has pointed out that the association of chemotherapy is, in general, more effective than RT alone in prolonging survival and in delaying recurrences in glioma patients. Particularly this meta-analysis showed a mild but significant benefit with the addition of CT, with a 15% relative reduction in the risk of death and an increase in 2-year survival from 9% to 13% in individuals with GBM and from 31% to 37% in pts with AA. These evidences encouraged research with new chemotherapeutic agents.

Temozolomide (TMZ) (Temodal, Temodar; Schering-Plough, Kenilworth, NJ) is one of second-generation imidazotetrazinone prodrugs that spontaneously converts into the active metabolite without the need for enzymatic demethylation in the liver [8]. Nowadays TMZ is the first choice drug in the chemotherapy of gliomas and is largely used after surgery and together or after RT.

Accordingly, 64 consecutive pts, with diagnosis of high grade glioma, with irradiation and adjuvant TMZ (group A, *n* = 31 pts) or adjuvant/concomitant (group B, *n* = 33 pts) [9] were treated at the Departments of Radiotherapy of Bari University and “Casa Sollievo della Sofferenza” Hospital in San Giovanni Rotondo. The survival data of these subjects (groups A + B) were compared with a group of 64 other patients with similar clinical characteristics treated in the same institutions only with radiotherapy from April 1994 to December 1996 (group C).

This study is aimed at comparing the outcome of the 31 pts treated with RT and adjuvant TMZ (group A) from January 1997 to June 1999 versus the 33 pts treated with RT and concomitant TMZ (group B) from July 1999 to November 2001 and at comparing, on a long term basis, the subjects that received both RT and TMZ with the historical group (group C) that was treated with radiotherapy alone. The survival data have been evaluated also according to the histology of the neoplasm: glioblastoma multiforme (GBM, in 43 patients of groups A + B and in 50 pts of group C) and anaplastic astrocytoma (AA, in 21 pts of groups A + B and in 14 pts of group C).

## 2. Patients and Methods

### 2.1. Inclusion Criteria

Our retrospective analysis included pts aged >18 years with pathologically proven diagnosis of AA or GBM. All histologic specimens were classified according to World Health Organisation (WHO) criteria, after surgery or stereotactic biopsy. Other inclusion criteria were a Karnofsky Index (KI) of 60–100, normal haematological, renal, and hepatic functions, absence of previous (with the exception of nonmelanoma skin cancer and carcinoma in situ of the cervix) or concurrent neoplasm, and absence of any other remarkable disease.

### 2.2. Patients' Characteristics

The study refers to 64 consecutive pts referred to our Departments of Radiotherapy that started brain neoplasm treatment from January 1997 to November 2001.

Out of 64 pts, 29 were females and 35 males, with age ranging from 26 to 78 years, with a median of 59 years. In 12 pts, with inoperable diseases, only a stereotactic biopsy was performed and in 52 pts a surgical resection was performed (30 subtotal and 22 total) (Tables 1 and 2).

The histology of the neoplasm was glioblastoma multiforme (GBM, in 43 patients of groups A + B) and anaplastic astrocytoma (AA, in 21 pts of groups A + B). The control group histology (group C) included 50 pts with GBM and 14 pts with AA.

### 2.3. Treatment

The 64 pts of groups A + B were treated with RT and oral TMZ. Median total dose of RT delivered was 63.5 Gy (range 45 Gy–66 Gy), with conventional fractionation, according to ICRU recommendations (Table 1). Ten pts were treated with a total dose less than 60 Gy because of their low (60 to 75) KI or disease progression during the treatment.

Irradiation volume was determined on preoperative computed tomography (CT) and magnetic resonance (MR) of the brain and planning target volume (PTV) included the neoplasm, the surrounding oedema, and a margin of 2 cm in all directions [10]. All pts were immobilised with a customised thermoplastic mask. Three-dimensional treatment planning was obtained on the basis of CT performed with pt immobilised in therapy position.

The first 31 pts from January 1997 to June 1999 (group A) were treated with RT and adjuvant TMZ (200 mg/m^2^/d × 5 days, every 28 days for 6 cycles) and the other 33 from July 1999 to November 2001 (group B) with RT and concomitant TMZ (75 mg/m^2^/d × 7 d/wk for 6 weeks) followed by adjuvant TMZ (200 mg/m^2^/d × 5 days, every 28 days for 5-6 cycles).

During the concomitant and adjuvant radiochemotherapeutic regimens, prophylactic antiemetic therapy (Ondansetron 8 mg/die or Granisetron 2 mg/die) was routinely prescribed. Anticonvulsant and corticosteroids were used only as required.

The results obtained in the 31 pts of group A have been compared with those of the 33 of group B. Finally we have compared the results obtained in these 64 pts (group A + group B) with those of a historical group of 64 consecutive pts (group C), with similar clinical characteristics, treated with RT alone at the same institutions from April 1994 to December 1996 (Table 2).

### 2.4. Statistical Analysis

Survival was calculated actuarially using the Kaplan Meier method, and significance was assessed using the log-rank test. Multivariate analysis by the Cox Regression Model was performed for identifying the independent prognostic variables governing the clinical end points.

The length of survival was considered from the end of radiation treatment until the last follow-up or the death.

## 3. Results

### 3.1. Survival

At the time of this analysis, May 2014, 3 pts (9.6%) in group A with AA and 5 pts (15.1%) in group B with AA are still alive, and 56 pts are dead (28 pts in group A and 28 pts in group B). The median follow-up in group A pts has been 18 months (range 6–89) and in group B 16 months (range 3–70).

**Figure 1:**
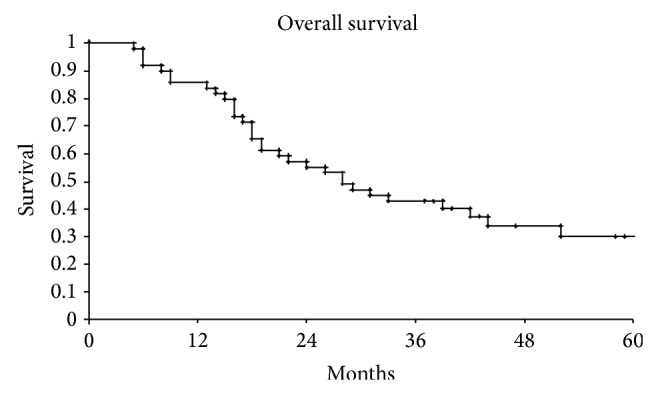
Overall survival of our series.

On the basis of Kaplan Meiers estimates, the 1- and 2-year overall survival rates (OS) were, respectively, 74% and 29% in group A pts and 73% and 30% in group B (*P* = 0.8 not statistically significant) (Figure 1).

On the contrary, a statistically significant better 2-year OS was observed in pts with age ≤55 years (*P* = 0.04) and/or with diagnosis of AA (*P* < 0.0001) and/or with total dose delivered ≥60 Gy (*P* = 0.001) (Table 4).

The multivariate analysis, using stratified Cox regression, disclosed a significant better 2-year OS associated with age ≤55 years (*P* = 0.04), diagnosis of AA (*P* = 0.0003), and type of surgery (*P* = 0.05). Timing of TMZ administration (group A versus group B) was not statistically significant (Table 5).

**Figure 2:**
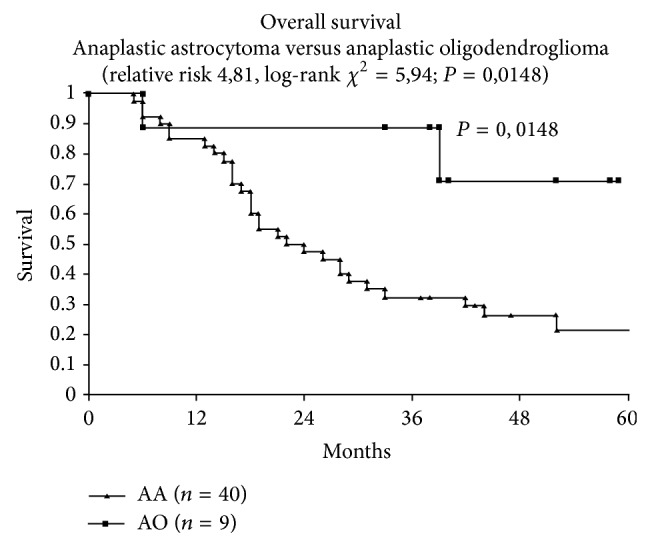
Overall survival anaplastic astrocytoma versus anaplastic oligodendroglioma.

Comparing the results of the 64 pts of groups A + B versus the 64 pts of group C, the median follow-up in groups A + B pts has been 17.5 months (range 3–89) and in group C pts 14 months (range 4–62). On the basis of Kaplan Meier estimates, the median OS was 15 months: 14 months in subjects not treated with TMZ (group C) and 17.5 months in the patients (groups A + B) that received TMZ (*P* = 0.0001) (Figure 2). Age, AA histology, and administration of TMZ were statistically significant prognostic factors for 2-year OS in the univariate analysis using Kaplan Meier method and compared with log-rank test: age ≤ 55 years *P* = 0.007; AA histology *P* < 0.0001; administration of TMZ *P* = 0.0001 (Table 6).

PFS was for GBM 9 months and for AA 11.

The salvage therapies employed in local recurrence are fotemustine, antiangiogenic drugs, and temozolomide.

### 3.2. Toxicity

We analysed complications of the 64 pts treated with RT and TMZ according to the WHO-RTOG scale. Grade 3 (G3) haematological toxicity was scored in 6 pts (9% of pts): 2 belonging to group A and 4 to group B (*P* = 0.6 not statistically significant) (Table 3).

In group A pts, during adjuvant chemotherapy, only one patient developed G3 neutropenia-thrombocytopenia and a further subject showed G3 neutropenia alone. No pts of group A experienced thrombocytopenia alone. In the subjects treated with concomitant TMZ and RT (group B) we observed G3 neutropenia in 2 cases, G3 thrombocytopenia in 1, and neutropenia and thrombocytopenia in a further *r* patient (Table 3).

No G4 haematological toxicity was observed.

The other acute side effects (G1-G2 nausea, vomiting, and fatigue), reported in 10 pts, of groups A + B were mild or easily controlled with medications.

## 4. Discussion

Malignant gliomas are among the most uncontrollable, devastating, and fatal cancers. The benefit of RT alone, in inoperable pts, or in combination with surgery, has been demonstrated in phase III trials at the end of seventies [11–14]. In order to improve the outcome, various combinations of surgery, RT, and chemotherapy have been tried in several studies, unfortunately with inconclusive results [1–6]. A meta-analysis [7] has pointed out significant improvement in survival adding nitrosourea based regimens.

TMZ is nowadays the first line chemotherapeutic drug in GBM therapy. Our study confirms its usefulness and the lack of heavy side effects.

Experimental studies demonstrated in vitro synergistic effect, in inhibiting glioblastoma cell lines growth, by using TMZ and fractionated RT [15]. On the basis of these suggestions, phase I and II clinical trials investigated the efficacy of this association, with promising results [16–24].

According to these encouraging experiences in 1997 we started to treat pts affected by high grade gliomas with a combination of TMZ and RT. The results of our study confirm the literature data regarding tolerability and usefulness of this schedule.

The main toxicity in our experience has been haematological, with G3 neutropenia, thrombocytopenia, or both observed in 9% of cases and above all in concomitant/adjuvant TMZ administration, without statistical significance. Similar incidence of haematological toxicity has been reported in Stupp phase II trial [22] and in other preliminary experiences [16, 25]. In our series we observed neither G4 haematological side effects nor infections of* Pneumocystis carinii* [17, 22]. Moreover nonhaematological toxicities were mild and easily controlled by medical therapies.

The median survival obtained in our pts is similar to that reported in other publications [26–30]. Two-year OS in groups A + B pts was 29.6% but 9.3% in group C (*P* = 0.0001), suggesting a significant improvement of prognosis by combined treatment. Similar results, using RT and TMZ, have been shown in other phase II trials with 2-year survival ranging from 29% to 38% [26–30]. Moreover in the multicentric randomized EORTC-NCIC 26981 trial 2-year survival was 26% in the 287 pts of RT + TMZ arm versus 8% in the 286 pts of RT alone arm (*P* < 0.0001) [24, 31].

In our pts no statistically significant difference in OS between adjuvant and concomitant/adjuvant TMZ administration was observed. Anyway preclinical studies and larger clinical trials have suggested additive or perhaps synergistic activity combining TMZ and RT [22].

In agreement with literature [22, 32, 33], our data confirm that age and histology represent important prognostic factors in this disease. In fact both univariate and multivariate analyses showed that pts with age ≤55 years and diagnosis of anaplastic astrocytoma have a significantly better survival.

Improvement of prognosis, obtained using RT and TMZ in malignant gliomas in several phase II series and above all in the phase III EORTC-NCIC 26981 trial on GBM, suggests that actually this treatment can be used routinely in clinical practice [24].

Despite these interesting results the prognosis of malignant gliomas remains poor. Concerning this, great advances could come from research into genetic features of brain tumours, with the aim of characterising molecular profiles of neoplasm [34]. These developments will identify novel drug targets and therapeutic strategies, in order to individuate subgroups of pts receiving tailored treatments, on the basis of the genetic findings of their cancers [34]. According to these remarks, some recent reports show the preliminary results of combining TMZ with other drugs active against biological targets, particularly antiangiogenic drugs like thalidomide [35] and rofecoxib [36] and other proposed “old” drugs like metformin and arsenic trioxide [37].

In order to increase the efficacy of TMZ, Brock et al. [35] have employed in 67 pts with glioblastoma an association of TMZ, thalidomide, and RT. They observed an acceptable tolerance and a favourable survival outcome when compared with a historical group of pts treated with RT alone or RT and nitrosourea adjuvant chemotherapy.

Similar findings were reported by Baumann et al. [36] in a recent publication, whereas in a preliminary study TMZ was tested in pts with GBM in combination with the COX-2 inhibitor rofecoxib, another antiangiogenic agent, in order to evaluate the safety and activity of this association [38].

Moreover several phase I and II trials are exploring possible therapeutic approaches with schedules containing TMZ and new drugs [23, 39, 40].

Some authors believe that the additions of TMZ do not change the pattern of progression of GBM after radiotherapy (GUNJUR A. J. M. I. and RADIATION ONCOLOGY 2012).

On the contrary, the majority, considering that patients aged 75 or older represent half of all patients with GBM, retain that older cohort (>65 years) should not be excluded from treatments as was shown in NOA-8 phase III study; data from randomized and nonrandomized studies show encouraging results.

Finally, elderly patients will soon represent the vast majority of patients with GBM and they deserve to be treated at the best way possible; future studies should include the older patients with stratification of comorbidities and PS.

For what concerns anaplastic astrocytomas, the treatment of anaplastic glioma varies depending on histopathology of the tumor, molecular markers, and individual patient characteristics. As opposed to the standard treatment of glioblastoma, based on Stupp trial, there is no accepted standard treatment for AG. AA is most often treated with radiotherapy, with or without concomitant TMZ and with or without adjuvant temozolomide. Temozolomide has largely replaced PCV (procarbazine, CCNU, and vincristine) as the chemotherapeutic agent for AO and AOA, largely due to greater tolerability and less potential for toxicity. However, whether temozolomide has similar efficacy to PCV has not been fully evaluated. Patients, who have progressed after RT alone, may be treated with TMZ or PCV. A valid option, at the recurrence, is stereotactic radiosurgery and we employ this modality in many patients.

In conclusion there is, today, an improvement in surgical techniques, such as fluorescence guided resection and neuroendoscopic approaches; new discoveries will be made in basic and translation research, with block of cancer proliferation (e.g., TMZ, BEVACIZUMAB, IHDAC, anti-P53 inhibitors, inhibition of cancer stem cells, more advanced and precise radiation techniques, inhibitors of EGFR, TKI, NF-KB, inhibitors of mTOR, Pi 3k/AKT, and proteasome) and new delivery of drugs in nanoparticles and liposomes and the introduction in clinical practice of antipsychotic drugs (like haloperidol of II and III generation). All that will, probably, improve survival and quality of life in such a devastating disease.

## 5. Conclusions

Continuous daily TMZ and concomitant RT followed by adjuvant TMZ are safe and can prolong survival in pts with high grade gliomas. Our report confirms the beneficial role of the association RT + TMZ on long (>10 y) follow-up. It must be stressed that this association resulted in life-saving on a 13-year time span in 3 out the 21 patients (14.1%) with anaplastic astrocytoma.

There is today a large interest in new treatments in gliomas like improvement in surgical techniques such as fluorescence guided resection and neuroendoscopic approaches. New discoveries are made in basic and translation research, and old and new drugs have been proposed as promising agents in brain tumors care [41–50]. More advanced and precise radiation techniques, inhibitors of EGFR, TKI, NF-KB, inhibitors of mTOR, Pi 3k/AKT, proteasome, and new delivery of drugs in nanoparticles, liposomes, and the introduction in clinical practice of antipsychotic drugs (like haloperidol of II and III generation) could be beneficial. All these improvements and developments will, probably, improve survival and quality of life in such a devastating disease.

## Figures and Tables

**Table 1:** Group A and group B: patients' characteristics.

	Group A	Group B
Patients' number	31	33
Age		
Range	41–78 years	26–74 years
Median	62 years	57 years
Sex		
Male	17 pts	18 pts
Female	14 pts	15 pts
Karnofsky Index		
Range	60–90	60–90
Medium	70	70
Surgery		
Stereotactic biopsy	3 pts	9 pts
Subtotal resection	18 pts	12 pts
Total resection	10 pts	12 pts
Histology		
Anaplastic astrocytoma	11 pts	10 pts
Glioblastoma multiforme	20 pts	23 pts
RT total dose		
Median	64 Gy	63 Gy
Range	45 Gy–66 Gy	45 Gy–64 Gy
<50 Gy	2 pts	1 pts
≥50 Gy <60 Gy	5 pts	2 pts
≥60 Gy	24 pts	30 pts

**Table 2:** Groups A + B and group C: patients' characteristics.

	Groups A + B	Group C
Patients' number	64	64
Age		
Range	26–78 years	29–74 years
Median	59 years	60 years
Sex		
Male	35 pts	39 pts
Female	29 pts	25 pts
Karnofsky Index		
Range	60–90	60–90
Medium	70	70
Surgery		
Stereotactic biopsy	12 pts	11 pts
Subtotal resection	30 pts	35 pts
Total resection	22 pts	18 pts
Histology		
Anaplastic astrocytoma	21 pts	14 pts
Glioblastoma multiforme	43 pts	50 pts
RT total dose		
Median	61 Gy	59 Gy
Range	45 Gy–66 Gy	35 Gy–66 Gy
<50 Gy	3 pts	3 pts
≥50 Gy <60 Gy	7 pts	6 pts
≥60 Gy	54 pts	55 pts

**Table 3:** G3 haematological toxicity in group A and group B patients.

	Group A	Group B
Patients' number	31	33
Haematological toxicity	6.4%	12.1%
Thrombocytopenia	0%	3%
Neutropenia	3.2%	6%
Thrombocytopenia-neutropenia	3.2%	3%

**Table 4:** Univariate analysis group A and group B patients.

Prognostic factors	Pts' number	2-year OS	*P*
Age			
≤55 years	26	38.4%	0.04
>55 years	38	23.7%	
Sex			
Male	35	28.6%	n.s.
Female	29	31%	
Histology			
Anaplastic astrocytoma	21	52.4%	<0.0001
Glioblastoma Multiforme	43	16.3%	
Surgery			
Stereotactic biopsy	12	25%	n.s.
Subtotal resection	30	23.3%	
Total resection	22	36.6%	
Timing TMZ			
Adjuvant	31	29%	n.s.
Concomitant/adjuvant	33	30%	
RT total dose			
≥60 Gy	54	33.3%	0.001
<60 Gy	10	10%	

**Table 5:** Multivariate analysis group A and group B patients.

Prognostic factors	*P*
Age	
≤55 years–>55 years	0.04
Sex	
Male—female	n.s.
Histology	
AA—GBM	0.0003
Surgery	
Stereotactic Biopsy—subtotal resection—total resection	0.05
Timing TMZ	
Adjuvant—concomitant/adjuvant	n.s.
RT total dose	
<60 Gy–≥60 Gy	0.0001

**Table 6:** Univariate analysis groups A + B and group C patients.

Prognostic factors	Pts' number	2-year OS	*P*
Age			
≤55 years	47	29%	0.007
>55 years	81	15%	
Sex			
Male	74	17.5%	n.s.
Female	54	22%	
Histology			
Anaplastic astrocytoma	36	40%	<0.0001
Glioblastoma multiforme	92	11.8%	
Surgery			
Stereotactic biopsy	23	25%	n.s.
Subtotal resection	65	14%	
Total resection	40	26%	
Treatment schedule			
RT + TMZ	64	29.6%	0.0001
RT alone	64	9.3%	
RT total dose			
≥60 Gy	109	22%	0.003
<60 Gy	19	10.5%	
